# Small Molecules Targeting HATs, HDACs, and BRDs in Cancer Therapy

**DOI:** 10.3389/fonc.2020.560487

**Published:** 2020-11-11

**Authors:** Donglu Wu, Ye Qiu, Yunshuang Jiao, Zhidong Qiu, Da Liu

**Affiliations:** ^1^ School of Clinical Medical, Changchun University of Chinese Medicine, Changchun, China; ^2^ Key Laboratory of Effective Components of Traditional Chinese Medicine, Changchun, China; ^3^ School of Pharmacy, Changchun University of Chinese Medicine, Changchun, China

**Keywords:** histone acetylation, cancer, histone deacetylase, histone deacetylase inhibitor, histone acetyltransferase

## Abstract

Evidence for research over the past decade shows that epigenetic regulation mechanisms run through the development and prognosis of tumors. Therefore, small molecular compounds targeting epigenetic regulation have become a research hotspot in the development of cancer therapeutic drugs. According to the obvious abnormality of histone acetylation when tumors occur, it suggests that histone acetylation modification plays an important role in the process of tumorigenesis. Currently, as a new potential anti-cancer therapeutic drugs, many active small molecules that target histone acetylation regulatory enzymes or proteins such as histone deacetylases (HDACs), histone acetyltransferase (HATs) and bromodomains (BRDs) have been developed to restore abnormal histone acetylation levels to normal. In this review, we will focus on summarizing the changes of histone acetylation levels during tumorigenesis, as well as the possible pharmacological mechanisms of small molecules that target histone acetylation in cancer treatment.

## Introduction

Histone post-translational modifications (PTMs) directly impact gene transcription by regulating the chromatin architecture (1). Histone acetylation is one of the most well-studied and important PTMs, which mainly affects the status of local chromatin relaxation through changing the distribution of histone acetylation marks in the local chromatin region, thereby regulating gene transcription activation (2). In more detail, the acetylation of histones occurs in the lysine residues on the N-terminal tail of the nucleosome histones composed of H2A, H2B, H3, and H4, and the histone deacetylases (HDACs) and the histone acetyltransferases (HATs) are responsible for adding or removing acetyl groups from the N-terminal tail of the nucleosome histones (3). A large amount of research data demonstrated that histone acetylation widespread in cells is involved in various cellular activities, including genome maintenance, biological processes, DNA damage repair, cell cycle, and apoptosis (4). Once the dynamic balance between acetylation/deacetylation in cells is disrupted, it will cause various diseases, such as Parkinson’s disease, leukemia, and even cancer (5–7). The following will specifically explain the changes in histone acetylation levels during cancer development, and how small molecules as cancer therapeutic drugs target and regulate intracellular acetylation levels.

## Imbalanced Histone Acetylation Levels in Tumorigenesis

**Figure 1:**
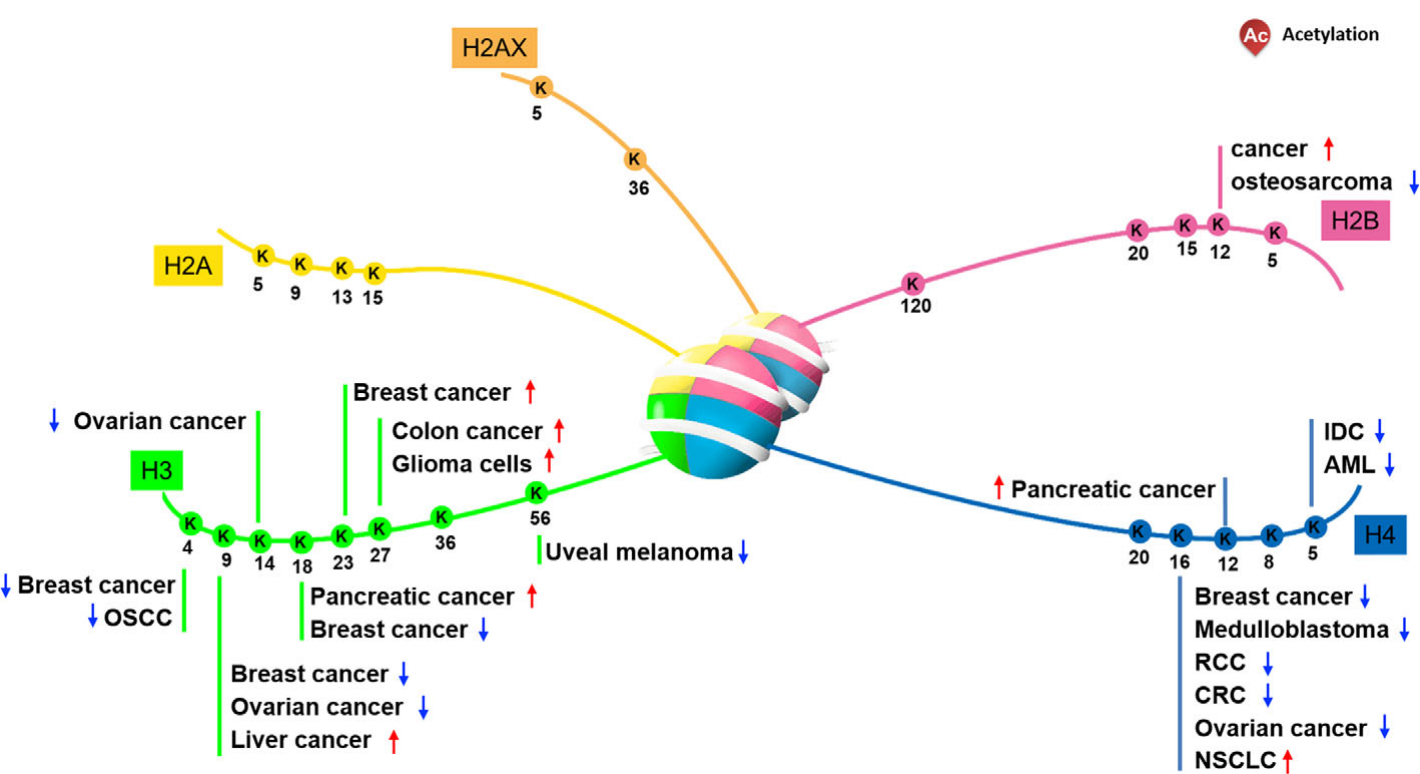
Aberrant acetylation on histone N-terminal sites in certain cancer. K, Lysine.

Based on the role of histone acetylation in the activation of gene expression, researchers speculated the mechanisms by which histone acetylation participated in and regulated progression of tumorigenesis (8). Multiple histone N-terminal acetylation sites have been identified (**Figure 1**). And many lysine sites on histones are obviously abnormally modified by acetylation in cancer cells and tumor tissues, suggesting that changes in their acetylation levels are closely related to the occurrence of cancer. Consistent with this argument, it has been confirmed that some HATs or HDACs are abnormally expressed when cancer occurs, resulting in alteration of local chromatin structure by changing the distribution of histone acetylation, ultimately affecting the expression of genes related to tumorigenesis.

It has been reported that the level of acetyl-modification on some histone lysine sites in cancer cells or tissues is obviously abnormal, and the increase or decrease of the modification level varies according to the type of cancer. Regarding H2A, *Hat1* knockdown- or *Tip60* abrogation-mediated downregulation of HeLa cell H2A lysine 5 acetylation (H2AK5ac) decreases HeLa cell colony size, suggesting that this acetylation can regulate cell proliferation (9). Furthermore, Ras-ERK1/2 pathway activation-induced osteosarcoma proliferation and migration co-occurs with downregulated H2BK12ac, a phenotype rescued by *HDAC1* knockdown-mediated H2BK12ac restoration (10). Relative to other types of histone acetylation, the H2BK20ac modification preferentially accumulates at promoters of cell type-specific genes, indicating a role in regulating cell-specific functions (11).

Previous data indicate that the acetylation of specific histone lysine sites is associated with the occurrence of certain cancers. Recent research reported that histone H3 acetylation level is correlated with the pathological stage of colorectal cancer, especially with the depth of tumor invasion (12). For instance, downregulation of H3K4ac and H3K9ac has been observed in oral squamous cell carcinoma and ovarian tumors, and the status of acetylation level is tightly correlated with tumor stage, perineural invasion and tumor prognosis (13–15). Part of the reason for the above results may be related to its distribution region on chromatin. Because subsequent studies found that H3K4ac is enriched in the promoter regions of genes which associated with cancer-related phenotypic features, such as the estrogen response and the epithelial-mesenchymal transition (EMT) pathway (16, 17). In head and neck squamous cell carcinoma (HNSCC) cells, H3K4ac modulated by HDAC3 is enriched around the transcription start site of EMT related genes such like GLI1 and SMO, co-overexpression of which promotes HNSCC cell invasion and migration ability (18). In addition to H3K4ac and H3K9ac, high-level of H3K23ac, which is correlated with TRIM24, has been observed in patients with HER2-positive breast cancer, and this correlates with a shorter survival interval (19). Moreover, H3K27 represents a site vulnerable to multiple modification types, including methylation and acetylation, and upregulated H3K27ac in colon cancer and glioma cells is correlated with tumor invasive capability (20, 21). In esophageal squamous cell carcinoma (ESCC), H3K27ac activates long non coding RNA colon cancer associated transcript-1 (CCAT1), thereby promotes ESCC cells proliferation and migration (22). It is worth noting that some lysine-sites acetylation on histone H3 have been used as biomarkers. For example, H3K18ac and H3K4me2 has been used as biomarker in prostate, pancreatic, lung, and kidney cancers (23, 24). Taken together, unbalanced acetylation level of histone H3 in various cancer tissues or cells suggests that H3 acetylation may be involved in the transcriptional regulation of cancer-related genes.

Regarding H4, modifiable residue K16 is well-studied, and H4K16ac is frequently downregulated in breast cancer, medulloblastoma (25, 26), renal cell carcinoma (RCC), colorectal cancer (CRC) (27, 28), and ovarian cancer (29, 30). However non-small cell lung carcinoma (NSCLC) exhibits upregulation of H4K16ac and HAT hMOF, resulting in downstream gene expression alterations correlating with tumor size, cell proliferation, and migration (31, 32). In particularly, in NSCLC cells hMOF promotes S phase entry by regulating Skp2, thereby stimulates NSCLC tumorigenesis (31). On the other hand, downregulation of H4K5ac observed in acute myeloid leukemia (AML) is associated with shorter survival intervals, and suppressed H4K5ac by MYST2 (Moz-Ybf2/Sas3-Sas2-Tip60) inhibition promotes AML cell growth and colony formation (33). In addition, downregulated H4K12ac consistent with HDAC1, HDAC2, and HDAC6 have been demonstrated *in situ* in invasive ductal carcinoma (34). Whereas upregulated H3K18ac and H4K12ac are observed in pancreatic cancer (24). A unique role for H4K20ac enriched at transcriptional start sites, co-localizing with NRSF/REST to participate in gene repression has been noted in cancer cells (35).

In summary, biological mechanisms employing acetylated histones are much more diverse than chromatin structure regulation alone. The numerous N-terminal tail lysine residue acetylation sites of H2A, H2B, H3, and H4 allow them to participate in various signaling pathways, and facilitate their multi-faceted roles in cancer cell biology. Indeed, various cancers exhibit a globally dysregulated histone acetylation pattern, correlating with progression, pathological stage, and prognosis. As such, acetylation patterns may have potential as valuable prognostic markers (24).

## HATs, HDACs and BRDs Act as “Writers”, “Erasers,” and “Readers” Respectively

Biological mechanisms employing acetylated histones are much more diverse than chromatin structure regulation alone. The numerous N-terminal tail lysine residue acetylation sites of H2A, H2B, H3, and H4 allow them to participate in various signaling pathways, and facilitate their multi-faceted roles in cancer cell biology. As mentioned, various cancers exhibit a globally dysregulated histone acetylation pattern, correlating with progression, pathological stage, and prognosis. As such, acetylation patterns may have potential as valuable prognostic markers (24). Noting that the dynamic change and reversible process of the acetyl-group at the N-terminal lysine site of histones can be controlled by certain proteins just like writers, erasers and readers. Cancer-associated abnormal histone acetylation profiles are due to corresponding aberrant expression or catalytic activities of these enzymes. HATs function as “writers”, transferring the acetyl group (-COCH_3_) from acetyl-CoA (Ac-CoA) to a target histone, whereas HDACs function as “erasers”, removing the acetyl group of a target histone (36, 37). However, whether it is to remove the acetyl group or recruit proteins to a specific acetyl-modified lysine site, the proteins usually have to recognize the acetyl group on a specific protein just like a reader.

**Figure 2:**
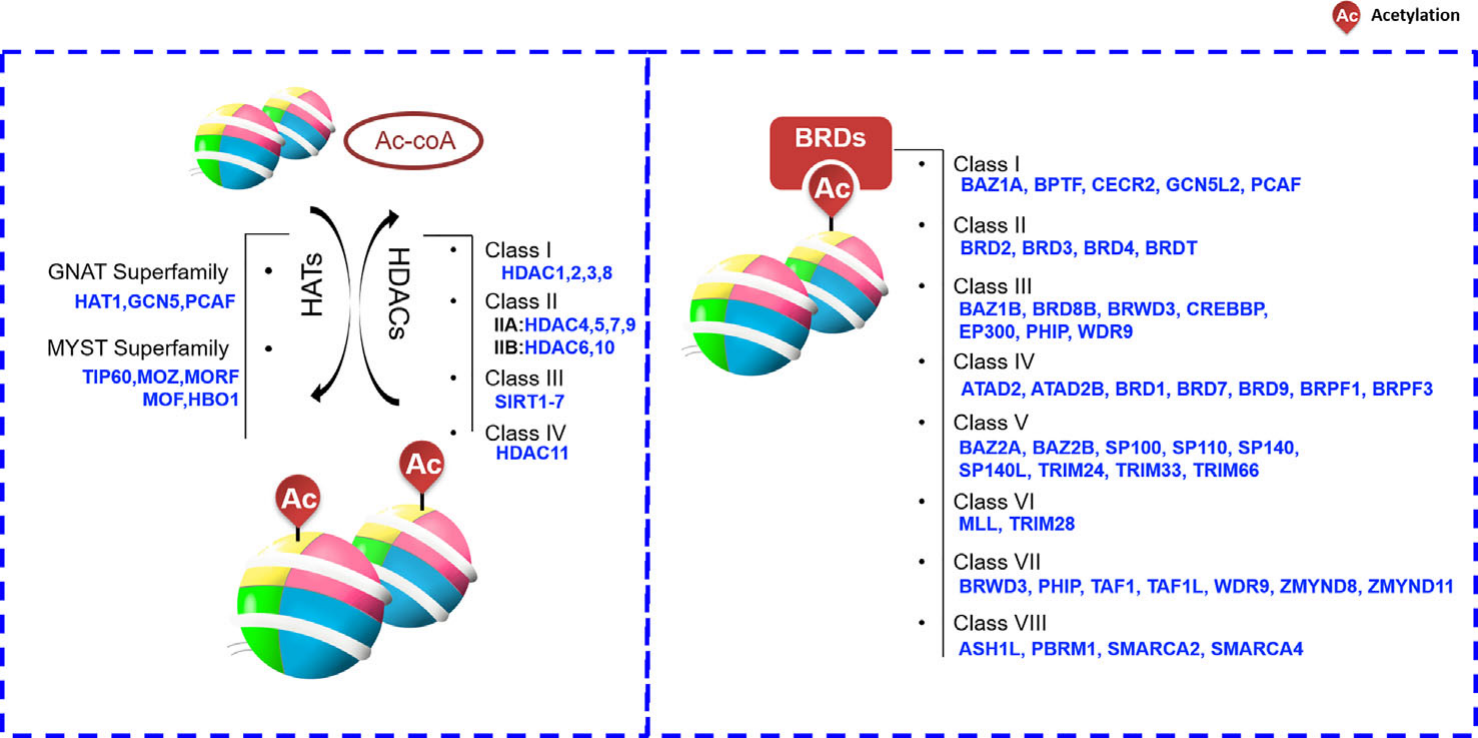
Histone acetylation “writers”, “erasers” and “readers”. ASH1L,ash1 (absent, small, or homeotic)-like; ATAD2, Two AAA domain containing protein; ATAD2B, KIAA1240 protein; BAZ, Bromodomain adjacent to zinc finger domain; BPTF, Fetal Alzheimer antigen; BRD, Bromodomain-containing protein; BRDT, Bromodomain-containing protein, testis specific; BRPF1, Bromodomain- and PHD finger-containing protein; BRWD3, Bromodomain-containing protein disrupted in leukemia; CBP, CREB-binding protein; CECR2, Cat eye syndrome chromosome region, candidate 2; CREBBP, CREB Binding Protein; EP300, E1A-binding protein p300; GCN5L2, General control of amino acid synthesis 5-like 2; GNAT, GCN5-related N-acetyltransferase; HAT, histone acetyltransferase; HDAC, histone deacetylases; MLL, Myeloid/lymphoid or mixed lineage leukemia; MYST, Moz-Ybf2/Sas3-Sas2-Tip60; ORPHAN, Orphan-containing family P300, E1A binding protein p300; PBRM1,Polybromo 1; PCAF, P300/CBP-associated factor; PHIP, Pleckstrin homology domain-interacting protein; SIRT, sirtuin; SMARCA, SWI/SNF-related matrix associated actin-dependent regulator of chromatin a; SP100, Nuclear antigen Sp100; SP110, Nuclear antigen Sp110 A; SP140, SP140 nuclear body protein; SP140L, SP140 nuclear body protein like; TAF1,TAF1 RNA polymerase II, TATA box-binding protein (TBP)-associated factor; TAF1L, TAF1-like RNA polymerase II, TATA box-binding protein (TBP)-associated factor; TIP60, Tat interactive protein 60-kDa; TRIM24, Tripartite motif-containing 24; WDR9, WD repeat domain 9; ZMYND8, Zinc Finger MYND-Type Containing 8; ZMYND11, remodeling factor containing 11.

Histone-mark readers often recognize marks through the functional domain contained in itself. Based on published literatures, the readers that can recognize histone acetylation are roughly divided into three categories including bromodomain-containing protein (BRD), PHD finger and YEATS domains. Among them, PHD finger and YEATS domain proteins have a wide range of functions. In addition to acetyl-group, they can also recognize methyl-group or other proteins. For example, PHD finger proteins can able to acquaint acetylated or unacetylated and methylated histones. However, BRD is the only protein group featuring a domain that is able to recognize and bind acetylated histone lysine residues. BRD-containing proteins are widely present in most tissues. According to the sequence or structure similarity, BRDs are divided into eight families exhibiting various activities, including histone modification and chromatin remodeling (**Figure 2**) (38, 39). For example, one of the most well-known BRD family members, BRD4, accumulates in highly acetylated and transcriptionally prone chromatin regions (including promoters and enhancers) and promotes RNA polymerase II (RNA Pol II) activity, thereby stimulating transcription initiation and transcript elongation. BRD4 is involved in HCC cell growth and invasiveness *in vitro*, and it is significantly upregulated in HCC tissue (a feature also associated with HCC progression) (40). Such functions are largely dependent on the ability of BRD4 to recognize acetylated proteins (41).

Considering the above description, the addition, removal and recognition of acetyl groups on histones is an indispensable dynamic balance. In other words, acetylation profiles regulated by HATs, HDACs, and BRDs, ultimately impact an abundance of target genes involved in tumorigenesis, thus regulating numerous cellular processes. For example, downregulation of TIP60 in 61% of primary gastric cancer patients is correlated with invasiveness and metastasis (42). Later research data supports this result. Currently, it is generally believed that alteration of HATs or HDACs level is involved in the occurrence and progression of cancer. From the published literature, the decrease of HATs and its enzymatic activity or the excessively high activity of HDACs can directly or indirectly affect the global acetylation level in cells. HAT MOF expression is downregulated in numerous cancers, including RCC, ovarian cancer, gastric cancer, and CRC (33). For additional detail, accumulating data reveals mutation residues on HATs in certain cancer, such as TIP60 in CRC (8). On the contrary, higher level of HDACs such as SIRT1, SIRT2, and SIRT7 were detected in cancer cells (43–45). Given this close relationship, an increasing number of small molecules targeting histone acetylation-regulating proteins are being investigated for their anti-cancer therapeutic potential.

## Small Molecules Targeting HATs, HDACS, and BRDs in Cancer Therapy

### HDAC Inhibitors (HDACis)

HDACs are enzymes that remove acetyl group on Lys residues of histone proteins, the following four classes of HDACs are recognized: I (HDAC1, 2, 3, and 8), II (A: HDAC4, 5, 7, and 9; B: HDAC6 and 10), III (SIRT1-7), and IV (HDAC11) (**Figure 2**) (46). Given that the HDACs frequently show higher expression levels in cancer cells, small molecules targeting HADCs were first investigated. At present, many small molecules have been developed as HDAC inhibitors (HDACis). These HDACis may target different stages of cancer or different signaling pathways, and ultimately achieve the purpose of inhibiting or treating cancer.

So far, five HDACis Vorinostat (SAHA), Belinostat (PXD-101), Panobinostat (LBH589), and chidamide (CS055, HBI-8000) and Romidepsin (FK228) have been approved by the U.S. FDA (Food and Drug Administration) as medicines for treatment of skin T-cell lymphoma (TCL) and peripheral TCL (47, 48). The former three HDACis inhibit class I, II, and IV HDACs, while Romidepsin selectively targets class I (47). As one of the best-studied and pan-HDACi SAHA induces autophagy of chronic lymphocytic leukemia, breast cancer as well as colon cancer cell lines, and the induced autophagy modulates mutant p53 degradation, further affects cancer cell survival (49, 50). In addition to use alone, SAHA induces radio treatment pancreatic cancer cell cycle arrest and apoptosis by targeting RAD51, clarifying the function of SAHA in enhance radiosensitivity (51). In combination with other anti-cancer drugs, such as oxaliplatin (Eloxatin) and ruxolitiniband, SAHA optimally inhibits cancer cell proliferation (52, 53). In addition, an isotretinoin-SAHA combination for the treatment of neuroblastoma is currently undergoing phase I clinical trials (54).

In addition to SAHA, there are already more than 20 kinds of HDACis are in different stages of clinical research, indicating that the research and development of HDACis is very popular and has broad development prospects. Most of the HDACis studied extensively are aimed at the proliferation of tumor cells by targeting cell cycle and apoptosis, growth, and migration capability (55). CG200745, is a pan HDACi, targets HDACs and modulates acetylation, thereby regulates down-stream genes including p53, myeloid cell leukemia-1 (Mcl-1) and B-cell lymphoma-extra large (Bcl-xL) (56, 57). In detail, CG200745 inhibits NSCLC cell growth by modulating the profile of H4K16ac at the transcription start site of cell proliferation related genes (58). Moreover, CG200745 (59, 60) enhances the expression of p53 target genes by regulating p53 acetylation, thereby inducing clonogenic cell death (56). (61) In pancreatic cancer, CG200745 elevates the H3 acetylation level and induces the expression of apoptotic proteins, furthermore, CG200745 works better in combination with gemcitabine or erlotinib in suppressing cancer cell proliferation (62). The ability of CG200745 to sensitize tumor cells to existing chemotherapeutic drugs (such as 5-fluorouracil (5-FU), cisplatin, and oxaliplatin) has also been demonstrated (57, 62–64). These data recommend the pan-HDACi CG200745 as a candidate anti-tumor drug or chemotherapy adjuvant, and is currently undergoing the phase I/II clinical trials for pancreatic cancer (62, 65–67).

Although the aforementioned pan-HDACis were approved for clinical application, side effects of these drugs like fatigue, nausea, thrombocytopenia, and cardiotoxicity limit its application (67). Thus, selective HDACis that target HDAC6, SIRT1 and SIRT2 have also appeared in recent years. For example, at least six HDAC6-selective inhibitors including SKLB-23bb, ACY1215 (rocilinostat), ACY241, Tubacin, Tubastatin A, and C1A have been reported (68). In several types of cancer cells such as bladder cancer, malignant melanoma and glioblastoma, HDAC6 is frequently over-expressed (69–71). As a mysterious of HDAC family, HDAC6 possess two catalytic domains and a ubiquitin-binding domain (BUZ), and selective-HDAC6 inhibitors are designed to block the effects of those special functional domains. Selective HDAC6 inhibitors Tubacin and tubastatin A are first developed because they can inhibit the proliferation of glioma and NSCLC by inhibiting autophagy and mediating the Notch1 signaling pathway (72, 73). Further research found that tubastatin A suppresses the ability of colony formation and migration, while in combination with temozolomide, tubastatin A accelerates glioblastoma cells apoptosis, and help glioblastoma multiforme cells overcome ER stress-tolerance (60, 74). Subsequent developed highly selective HDAC6 inhibitors including J22352, ACY1215 (Ricolinostat) and its analogue ACY241, JW-1, ACY1083 etc. come out one after another. Those small molecules present highly effective anti-cancer effects. Among them, ACY1215 and its analogue ACY241 appeared a good anti-tumor effect in synergy with other drugs (59, 61, 75). In particular, ACY1215 has already entered phase II treatment of multiple myeloma (76, 77), and ACY241 has been completed the phase I clinical trial in combination with paclitaxel in solid tumor models (66). In fact, more compounds are still in the experimental research stage. For example, J22352 as a highly HDAC6-selective inhibitor suppresses the proliferation as well as migration of glioblastoma through promoting the proteolysis degradation of HDAC6 and resulting in anti-cancer effect by inhibiting autophagy (71). It is worth noting that HDAC6 is a microtubule-associated deacetylase, which mediates microtubule-dependent cell motility (78, 79). HDAC6 inhibitors JW-1, ACY1083 as well as tubastatin A anchor this characteristic of HDAC6. By inhibiting HDAC6, they can promote the acetylation of α-tubulin (80–82) thereby regulating cancer cell cycle and proliferation (74, 83, 84). HDAC6-selective inhibitor C1A exhibits an additional mechanism of action, inhibiting neuroblastoma and CRC xenograft growth through the modulation of autophagy substrates (85). While MPT0G211 targets HDAC6 thereby accelerates the acetylation of heat shock protein 90 (Hsp90), further inhibits breast cancer metastasis (80). In combination with other anticancer drugs, HDAC6 inhibitor A542 suppresses the proliferation of follicular lymphoma (FL), chronic lymphocytic leukemia (CLL), germinal center diffuse large B-cell lymphoma cells (DLBCL) and CRC by targeting HDAC6 (86, 87). Furthermore, HDAC6 inhibitors such as JOC1, SKLB-23bb, MPT0G413 as well as MPT0G612 show great anticancer activity, whereas the cytoplasm toxic as well as the mechanism are to be further investigated (68, 88–91).

Sirtuins (SIRT1-7) are human homologs of the yeast Sir2 (silent information regulator-2) protein and are divided into four main classes: SIRT1-3 are class I, SIRT4 is class II, SIRT5 is class III and SIRT6-7 are class IV (92). SIRT proteins belong NAD-dependent deacetylases that act as intracellular regulators and are thought to have ADP-ribosyltransferase activity (93). It has been reported that (94–97) SIRT1 and SIRT2 as deacetylases modulate the acetylation of p53, thereby regulating p53 target genes and cancer cell progression (81, 98). JQ-101, which inhibits SIRT1-mediated H4K16 and p53 acetylation, thereby inducing A549 cell senescence and inhibiting tumor growth and invasiveness, similar phenomenon and mechanism has been detected in SIRT1 specific inhibitor EX527 treated glioma cells (82, 99). Moreover, AEM1 and AEM2 also can facilitate p53 acetylation by targeting SIRT2 and further regulating the expression of p53 target genes (e.g., cell cycle regulator p21), thereby sensitizing NSCLC cells to genotoxic stress (100). However, tenovin-6 modulates the mRNA and protein level of p21 in cancer cell lines but through a p53-independent mechanism (101–104).

Recently, with the development of HDAC inhibitors, many newly synthesized, derived derivatives or modified compounds have come out, and pre-clinical experiments have begun. For instance, a novel HDACi (OH-VPA) was developed by modifying a traditional HDACi (VPA), representing a new approach to novel HDACi development. The derivative HDACi is more effective in inhibiting HeLa cell proliferation than its parent molecule (105). In addition, many compounds are still in pre-clinical development, such as abexinostat, AR-42, chidamide, CHR-3996, CI-994, CUDC-101, CUDC-907, entinostat (MS-275), givinostat, MGCD0103, mocetinostat, phenylbutyrate, pivanex, pracinostat, quisinostat, ricolinostat, valproic acid (VPA). Some confer added benefits in combination with other drugs and are undergoing phase I/II clinical trials (**Table 1**) (62, 65, 94–97, 102–104, 106–109, 112–120, 123–130, 132–135, 139–143, 175, 176).

**Table 1:** Selective HDAC inhibitors in clinical trials (completed) (from clinicaltrials.gov as of October 2020).

Compound	HDAC Selectivity	Clinical Trial Phase and Indication(s)	ID# of clinical trial	Reference(s)
**Abexinostat**	Class I, II	Phase I for advanced solid tumors.	NCT01543763	(106, 107)
		Phase I/II for Hodgkin lymphoma.	NCT00724984	
		Phase I/II for non-Hodgkin lymphoma.	NCT04024696	
		Phase I/II for chronic lymphocytic leukemia.	NCT00724984	
		Phase I, combined with doxorubicin, for metastatic sarcoma.	NCT01027910	(108)
***ACY241***	HDAC6	Phase I, in combination with paclitaxel in patients with advanced solid tumors	NCT02551185	(66)
		Phase I, in combination with ipilimumab and nivolumab to patients with advanced melanoma.	NCT02935790	–
**AR-42**	Class I, IIb	Phase I for multiple myeloma and T- and B-cell lymphomas.	NCT01129193	(109)
		Phase I, in combination with decitabine in AML in adults and children.	NCT01798901	(110, 111)
**Belinostat**	Class I, II, IV	FDA approved for peripheral T-cell lymphoma.	NCT00865969	(94–97, 112, 113),
		Phase I/II for lymphomas and solid tumors.	NCT01273155	
		Phase I, combined with cisplatin and etoposide, for solid lung tumors.	NCT00926640	(114–117),
		Phase I/II, combined with doxorubicin, for soft tissue sarcomas.	NCT00878800	
		Phase II, combined with paclitaxel/carboplatin, for carcinoma.	NCT00873119	
		Phase I/II, combined with cisplatin, doxorubicin, and cyclophosphamide in thymic epithelial tumors.	NCT01100944	
**Chidamide**	HDAC1-3,10	Phase II, combined with paclitaxel and carboplatin for advanced NSCLC.	NCT01836679	(118)
**CHR-3996**	Class I	Phase I for refractory solid tumors.	NCT00697879	(102)
**CI-994**	Class I	Phase II, with or without gemcitabine for pancreatic cancer.	NCT00004861	(103)
		Phase II for myeloma.	NCT00005624	(104)
		phase III with or without gemcitabine for advanced NSCLC.	NCT00005093	(119)
**CUDC-101**	Class I, II HDAC/EGFR/HER2	Phase I for advanced solid tumors.	NCT00728793	(120)
		Phase Ib, for advanced head and neck, gastric, breast, liver, and non-small cell lung cancer tumors.	NCT01171924	(121)
		Phase I, in combination with concurrent cisplatin and radiation therapy in patients with locally advanced head and neck cancer.	NCT01384799	(122)
**CUDC-907**	Class I, II	Phase I for B-cell lymphoma.	NCT02674750	(123)
		Phase I, for advanced/relapsed solid tumors	NCT02307240	–
**Entinostat** **(MS-275)**	Class I	Phase I/II for RCC.	NCT03552380	(124, 125),
		Phase II for relapsed and refractory Hodgkin lymphoma.	NCT00866333	
		Phase II, combined with 5-azacitidine and entinostat, for advanced breast cancer and metastatic CRC.	NCT01105377	(126, 127),
		Phase I for advanced solid tumors or lymphoma.	NCT00020579	(128–130)
		Phase I/II, combined with avelumab for epithelial ovarian cancer.	NCT02915523	
		Phase I, combined with exemestane, for breast cancer.	NCT02833155	
		Phase II, combined with azacitidine, for metastatic CRC.	NCT01105377	
		Phase I/II, combined with azacitidine, for recurrent advanced NSCLC.	NCT00387465	
**Givinostat** **(ITF2357)**	Class I, II	Phase II, ITF2357 followed by Mechlorethamine administered to patients with relapsed/refractory Hodgkin’s lymphoma.	NCT00792467	- (131)
**Mocetinostat (MGCD0103)**	Class I, IV	Phase I for advanced solid tumors or Non-Hodgkin’s Lymphoma.	NCT00323934	(132)
		Phase I, combined with docetaxel for advanced solid tumors.	NCT00511576	(133)
		Phase II for relapsed/refractory lymphoma.	NCT00359086	(134, 135)
		Phase II, combined with gemcitabine, for metastatic leiomyosarcoma	NCT02303262	(133)
		Phase II, for advanced urothelial carcinoma.	NCT02236195	(136)
		Phase I/II, combined with durvalumab for advanced solid tumors and NSCLC	NCT02805660	–
		Phase II for refractory chronic lymphocytic leukemia	NCT00431873	(137)
		Phase I for leukemia.	NCT00324194	(138)
		Phase I/II, in combination with azacitidine for AML.	NCT00324220	–
		Phase I/II, combined with gemcitabine for solid tumors.	NCT00372437	(133)
**Panobinostat**	Class I, II, IV	FDA approved for multiple myeloma.	NCT02568943	(139–141)
		Phase II for lymphoma/waldenstrom macroglobulinemia.	NCT01261247	
		Phase I/II, combined with bortezomib, thalidomide, and dexamethasone, for relapsed multiple myeloma.	NCT01023308	(142)
**Phenylbutyrate**	Class I, II	Phase I for solid tumors or lymphomaa.	NCT00002909	(143)
		Phase I, combined with azacitidine, for refractory solid tumors.	NCT00005639	(144)
		Phase II, for brain tumors in children	NCT00006450	–
		Phase I, for brain neoplasms and neuroblastoma	NCT00001565	–
		Phase I, in combination with azacitidine for AML	NCT00004871	(145)
**Pivanex**	Class I, II	Phase II, in combination with docetaxel for advanced NSCLC.	NCT00073385	(146)
**Pracinostat** **(SB939)**	Class I, II, IV	Phase I, treatment alone or with azacitidine for advanced solid tumors.	NCT00741234	(147)
		Phase I, combined with azacitidine for AML.	NCT01912274	(148)
		Phase I, for locally advanced or metastatic solid tumors.	NCT00504296	–
		Phase I, for solid tumors and leukemia	NCT01184274	–
		Phase II, for recurrent or metastatic prostate cancer.	NCT01075308	(149)
		Phase II, for advanced or recurring sarcoma.	NCT01112384	(150)
**Quisinostat** **(JNJ-26481585)**	Class I, II	Phase I for advanced solid tumors and lymphoma.	NCT00677105	(151)
		Phase II, in combination with paclitaxel and carboplatin for advanced epithelial ovarian cancer, primarily peritoneal or fallopian tube carcinoma.	NCT02948075	–
		Phase II, for cutaneous T-cell Lymphoma.	NCT01486277	(152)
		Phase I, in combination with gemcitabine and cisplatin for NSCLC, in combination with paclitaxel and carboplatin for NSCLC and ovarian cancer	NCT02728492	–
		Phase I, in combination with bortezomib and dexamethasone for relapsed multiple myeloma	NCT01464112	(153)
**Ricolinostat** **(ACY1215)**	HDAC6	Phase Ib, ACY-1215 monotherapy in patients with lymphoid malignancies.	NCT02091063	–
		Phase Ib, combined with lenalidomide and dexamethasone for relapsed or refractory multiple myeloma.	NCT02189343	(154)
		Phase I and phase IIa, alone or in combination with bortezomib and dexamethasone in patients with relapsed or relapsed/refractory multiple myeloma.	NCT01323751	(155)
**Romidepsin** **(FK228)**	Class I	FDA approved for cutaneous/peripheral T-cell lymphoma.	NCT00007345	(47, 156)
		Phase I/II for Japanese patients with relapsed or refractory peripheral T-cell lymphoma.	NCT00426764	
		Phase I, combined with ifosfamide, carboplatin, and etoposide for relapsed or refractory peripheral T-cell lymphoma.	NCT01590732	(131, 157)
		Phase I/II, combined with erlotinib hydrochloride, for lung cancer and metastatic cancer.	NCT01302808	
		Phase I/II, combined with abraxane for metastatic inflammatory breast cancer.	NCT01938833	
		Phase I/II, combined with cisplatin and nivolumab, for triple negative breast cancer.	NCT02393794	
		Phase II for recurrent and/or metastatic thyroid cancer.	NCT00098813	
		Phase I, combined with gemcitabine for pancreatic cancer.	NCT00379639	
**Valproic Acid** **(VPA)**	Class I, II	Phase II for prostate cancer.	NCT00670046	(158–160)
		Phase II, combined with bevacizumab, mFOLFOX6/mOXXEL, Capecitabine,5-fluorouracil, for ras-mutated metastatic CRC.	NCT04310176	
		Phase I, combined with azacitidine, for advanced cancers.	NCT00496444	
		Phase I, combined with etoposide for neuronal tumors and brain metastases	NCT00513162	
**Vorinostat** **(SAHA)**	Class I, II, IV	FDA approved for cutaneous T-cell lymphoma.	NCT00958074	(161)
		Phase I, combined with isotretinoin, for refractory/recurrent neuroblastoma.	NCT01208454	(54, 162, 163)
		Phase II, combined with bevacizumab, for malignant glioma.	NCT01738646	
		Phase I/II, combined with bevacizumab and temozolomide, for recurrent malignant gliomas.	NCT00939991	
		Phase II, combined with MK0683 and vorinostat, for advanced cutaneous T-cell lymphoma.	NCT00091559	(164–166)
		Phase II for progressive metastatic prostate cancer.	NCT00330161	(167, 168)
		Phase II for progressive or recurrent glioblastoma multiforme.	NCT00238303	(169)
		Phase I/II for advanced BRAF mutated melanoma.	NCT02836548	(170)
		Phase II, combined with paclitaxel, carboplatin, placebo, for stage III or stage IV NSCLC.	NCT00481078	(171)
		Phase I/II, combined with pembrolizumab for squamous cell head and neck cancer or salivary gland cancer.	NCT02538510	(172)
		Phase I, combined with pazopanib for advanced cancer.	NCT01339871	(173)
		Phase I/II for multiple myeloma.	NCT00857324	(174)

AML, acute myeloid leukemia; CRC, colorectal cancer; NSCLC, non-small-cell lung cancer, RCC, renal cell carcinoma.

### Small Molecules Targeting HATs

This review limits its scope to discussing only HAT inhibitors which have been approved for cancer therapy or commercialization, since the specific mechanisms of HAT modulation-mediated anti-cancer effects are complex and ambiguous (177, 178). It appears that HAT influence during carcinogenesis is context-specific because HATs are able to act as both oncogenes and tumor suppressors (179). The possible reason is that different tumors show mutations in different HAT members, which directly or indirectly affects any steps in the continuous process of tumor progression from tumorigenesis to carcinogenesis and metastasis (180). Based on sequence homology and shared structural features, HATs can be divided into two different classes. One is the GCN5-related N-acetyltransferases (GNATs) family, including GCN5 and p300/CBP-associating factor (PCAF), that can acetylate lysine residues on histones and non-histone proteins (181). In lung cancer cells, p300 may promote Snail-dependent EMT (epithelial-mesenchymal transition) by acetylating Snail at K187 site (182, 183). At present, several small molecule compounds targeting p300 have been developed and proved to have anti-cancer effects. For example, (184) Garcinol facilitate HeLa cell apoptosis *via* inhibiting the HAT activity of P300 and PCAF (185). Similarly, the molecule PU141, a selective CBP/P300 inhibitor, suppresses murine SK-N-SH neuroblastoma xenograft survival (186). Another HAT inhibitor C646 suppresses gastric cancer cell survival and invasive capability through competitively disrupting the interaction between Ac-CoA and CBP/P300 (187, 188). Recently discovered compounds CCT077791 and CCT077792 were also found to target P300 and PCAF, and resulting in the reduction of global acetylation level in colon tumor cell acetylation levels and inhibiting tumor cell growth (189).

Another HAT family is the MYST superfamily, exhibiting a conserved catalytic MYST domain, and large group membership, including MOZ, Ybf2, Sas2, TIP60, and hMOF (181). The role of MYST family in tumorigenesis is beyond doubt. Based on laboratory research data, Tip60 can harbor substrates including histones and non-histone proteins like p53 and ATM kinase, through which TIP60 plays critical roles in regulating cancer progression such as cell cycle, invasiveness and metastasis in gastric cancer and breast cancer cells (190, 191). Importantly, changes of downregulation of TIP60 is correlated with overall survival of breast cancer patients (42, 192). In addition, by regulating PI3K/AKT pathway, Tip60 suppresses the proliferation and migration of cholangiocarcinoma (111, 193)Given the critical role of TIP60 (a HAT which forms part of the TIP60/NuA4 complex) in DNA damage repair, several TIP60 inhibitors have been investigated for their anti-cancer therapeutic potential, including TH1834, NU9056, and 6-alkylsalicylates. Indeed, TH1834 (which blocks the binding site of TIP60) disrupts DNA damage repair to induce breast cancer cell apoptosis (194), and NU9056 both inhibits prostate cancer cell growth and induces apoptosis (195). Similarly, frequent downregulation of MOF has been detected in numerous cancers, including RCC, ovarian cancer, gastric cancer, and CRC (33). Developed MOF inhibitor DC-M01-7 downregulates H4K16ac, inhibiting proliferation of human colon cancer (HCT116) cells (196). Furthermore, through the role of HATs in DNA damage repair, several novel HAT inhibitors sensitize cancer cells to the cytotoxic effects of radiation therapy, suggesting their potential as adjuvants in this context (197, 198). However, there are few reports on selective inhibitors targeting members of this family.

### BRD Inhibitors

It is common for both histone acetylation and BRDs to become dysregulated in cancer. Current BRD inhibitors (e.g., isoxazoles, purines, quinolinones, tetrahydroquinolines, naphthyridines, and acetylated lysine analogs) exhibit high affinity and specificity for the BET bromine domain (199). Both I-BET 151 and I-BET 762 down-regulate c-Myc transcription, result in inhibition of myeloma cell proliferation (177). Moreover, I-BET 762 suppresses pancreatic cancer cell proliferation (178), and I-BET 762 inhibits breast and lung cancer cell proliferation through cell growth arrest and immune modulation (200). Whereas another BRD inhibitor JO1, by competing with histone acetylated residues, releases BRD4 from chromatin, thereby modulating RNA-Pol II activity to regulate the transcription of key cancer-associated genes (201). In addition, JQ1 decreases the acetylation level and activity of mutant p53, inducing cell growth arrest and subsequent senescence in HNSCC (202). OTX015 (MK-8628, birabresib), one of BRD and extra-terminal domain inhibitors, exhibits antitumor activity in medulloblastoma, B-cell lymphoma, and lung cancer (179, 184, 203). In addition, BET inhibitors such like PLX51107 and NHWD-870 have been identified the activity of tumor proliferation suppression (204, 205). By targeting the interaction of BRDs and acetylated lysine residues on histone, BRD inhibitors modulate chromosome structure and cancer-associated gene expression including *c-Myc*.

## Conclusions and Perspectives

**Figure 3:**
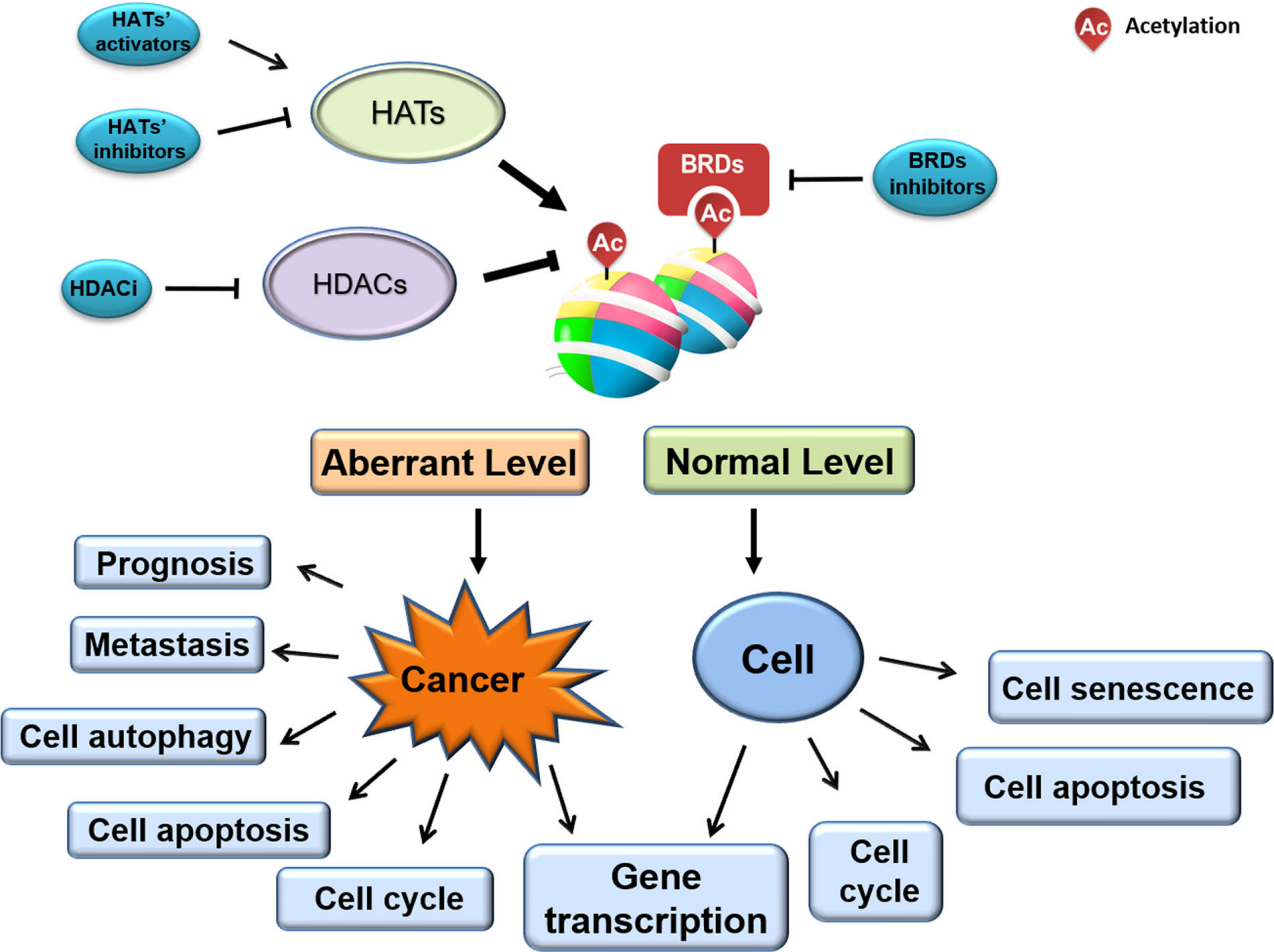
Links between histone acetylation level and cell cycle/cancer progression.

Altered histone acetylation—one of the earliest-identified and best-studied epigenetic modifications—is associated with tumorigenesis and tumor progression. Aberrant acetylation profiles are present across various cancer cells, tissues, and types. Given that dynamic histone acetylation/deacetylation is regulated by HDACs, HATs, and BRDs, many small molecules and novel synthesized compounds targeting enzyme catalytic activity or BRD/histone interaction are under investigation for their anti-cancer therapeutic potential (**Figure 3**). While several agents are already FDA-approved for clinical use, many more are undergoing clinical trials, and additional novel agents are being developed and tested. Indeed, the full clinical therapeutic scope and commercial value of such agents in the field of oncology is only just emerging.

## Author Contributions

ZQ and DL designed the review. DW, YQ, ZQ, YJ, and DL contributed to manuscript preparation. DW and YQ contributed equally. All authors contributed to the article and approved the submitted version.

## Funding

This work was supported by National Natural Science Foundation of China (Grant No. 81572868, 81803680, 81973712, 81903876). Jilin Scientific and Technological Development Program (Grant No. 20170309005YY).

## Conflict of Interest

The authors declare that the research was conducted in the absence of any commercial or financial relationships that could be construed as a potential conflict of interest.
